# Identification of PTPN1 as a novel negative regulator of the JNK MAPK pathway using a synthetic screening for pathway-specific phosphatases

**DOI:** 10.1038/s41598-017-13494-x

**Published:** 2017-10-11

**Authors:** Jiyoung Moon, Jain Ha, Sang-Hyun Park

**Affiliations:** Department of Biological Sciences, Seoul National University, Seoul, 08826 Korea

## Abstract

The mitogen activated protein kinase (MAPK) signaling cascades transmit extracellular stimulations to generate various cellular responses via the sequential and reversible phosphorylation of kinases. Since the strength and duration of kinase phosphorylation within the pathway determine the cellular response, both kinases and phosphatases play an essential role in the precise control of MAPK pathway activation and attenuation. Thus, the identification of pathway-specific phosphatases is critical for understanding the functional mechanisms by which the MAPK pathway is regulated. To identify phosphatases specific to the c-Jun N-terminal kinase (JNK) MAPK pathway, a synthetic screening approach was utilized in which phosphatases were individually tethered to the JNK pathway specific-JIP1 scaffold protein. Of 77 mammalian phosphatases tested, PTPN1 led to the inhibition of JNK pathway activation. Further analyses revealed that of three pathway member kinases, PTPN1 directly dephosphorylates JNK, the terminal kinase of the pathway, and negatively regulates the JNK MAPK pathway. Specifically, PTPN1 appears to regulate the overall signaling magnitude, rather than the adaptation timing, suggesting that PTPN1 might be involved in the control and maintenance of signaling noise. Finally, the negative regulation of the JNK MAPK pathway by PTPN1 was found to reduce the tumor necrosis factor α (TNFα)-dependent cell death response.

## Introduction

The mitogen-activated protein kinase (MAPK) pathways are evolutionally conserved among eukaryotes, and regulate diverse cellular responses, including cell proliferation, differentiation, and death^1,2^. Three kinases (MAPKKK, MAPKK, and MAPK) often form a signaling complex with a scaffold protein and relay phosphorylation in response to several extracellular stimuli, and thereby modulate MAPK signaling^3,4^. The magnitude and duration of MAPK activation have been shown to determine the resultant cellular response^5^. Furthermore, dysregulation of kinase function within the MAPK pathway has been demonstrated to lead to abnormal cellular responses and disease-like states^6^. Proper regulation of kinase activity within the pathway requires that several kinases, phosphatases, and scaffold proteins function and undergo complex interactions correctly.

The c-Jun N-terminal kinase (JNK) pathway is a major signal transduction pathway in mammalian cells, and comprises three kinases, MLK3 (MAPKKK), MKK7 (MAPKK), and JNK (MAPK)^7,8^. As an extracellular ligand, tumor necrosis factor α (TNFα) is the prototype for 20 related cytokines that stimulate both the JNK and NFκB pathways, and which also induce production of ROS^9,10^. Stimulation by TNFα leads to the activation of JNK signaling, in which MLK3 is recruited by TRAF2 and subsequently activated, as are both MKK4 and MKK7^11,12^. MKK7 activation induces the dual phosphorylation of JNK, and the cleavage of caspase 8 and Bid^13,14^. JIP1, a JNK pathway-specific scaffold protein, promotes efficient and precise JNK signal transduction by forming a signaling complex with the three kinases in response to extracellular stimuli. JNK pathway has been analyzed in various human disease states, e.g. cancer, diabetes, obesity, inflammatory diseases, and neurodegenerative diseases^15–18^. In most of those disease states, JNK pathway is dysregulated, often associated with upregulation of JNK activity resulting from malfunctioning of relevant phosphatases^19–21^. For example, when DUSP10, a negative regulator of JNK pathway, was deficient, it led to increased level of activated JNK and cytokine production^22^. Likewise, aging diabetic cell showed elevated JNK activity and reduced expression of DUSP10^17^. Proper regulation of JNK pathway by phosphatases, therefore, must be fulfilled to maintain normal physiology.

To elucidate the regulatory mechanisms of JNK MAPK signaling, it is essential to identify JNK pathway-specific phosphatases. Although there are more than 500 protein kinases identified in the human genome, only ~120 protein phosphatases have been discovered^23–28^. With a significant difference in the number of phosphatases compared to kinases, it is well known that phosphatases have multiple phosphoprotein targets^24,29–31^. This property of phosphatases may make it difficult to point out a phosphatase enzymatic activity toward a specific target among various phosphoproteins which often form complex crosstalk networks in the cell^32^. To clarify a specific substrate of a phosphatase within the pathway, we previously conducted a study that utilized a fusion protein model in which phosphatases were tethered to a scaffold protein (Ste5) of the mating signaling pathway in budding yeast^33^. In the present study, we used a synthetic approach to screen for JNK pathway-specific phosphatases. For the screen, the whole repertoire of phosphatases in cell were collected, and individual phosphatases were linked to JIP1 scaffold to increase stereophysical proximity between analyzed phosphatases and the pathway components complex with JIP1, thereby augmenting the effects of the analyzed phosphatases on JNK pathway activity. JIP1 has been previously shown to mediate TNFα-stimulation of the JNK pathway, such that TNFα treatment may be a useful means to stimulate JNK pathway for the screening that exploits JIP1-phosphatase tethers^13,14^. However, when expressed as a JIP1-phosphatase fusion protein, some phosphatases may inactivate the JNK pathway nonspecifically via enforced close proximity, and/or interrupt JNK signal transduction via disruption of the three-dimensional structure of JIP1 signaling complex. To exclude these potential false-negative effects, downregulation of JNK pathway by each analyzed phosphatase was confirmed by assessment of the co-expression of the corresponding phosphatase in its free form with JIP1. Together, this approach led to the identification of a novel phosphatase, non-transmembrane protein tyrosine phosphatase 1 (PTPN1).

PTPN1 was first discovered in isolates from human placental tissues^34^ and the three-dimensional structure of PTPN1 has been subsequently identified^35^. Previous studies have shown that PTPN1 directly dephosphorylates insulin receptor (IR) and insulin receptor substrates (IRS), which results in downregulation of insulin signaling^36,37^. For example, PTPN1 deficient mice show increased insulin sensitivity^38,39^. PTPN1 also dephosphorylates activated STAT3 and JAK2, and regulates leptin signaling^40–42^. PTPN1 contains a signature PTP family motif [I/V]HCXXGXXR[S/T] located beside the WPD loop, the Q loop, and the Tyr loop^43^. The WPD loop contains a conserved acidic residue (Asp181 in PTPN1) that plays an important role in protonation of the tyrosyl leaving group of a substrate. Mutation of Asp181 in PTPN1 results in the suppression of PTPN1 catalytic activity; however, the affinity of PTPN1 for substrates is maintained. Thus, PTPN1 D181A acts as a ‘substrate trap’ that exerts a dominant-negative effect^43,44^. The structural approach also provided further analysis of potential substrates of PTPN1 *in vivo* ^44^. With these studies, PTPN1 has become an attractive therapeutic target of diabetes and obesity. In the present study, PTPN1 was shown to reduce TNFα-stimulated cell death responses via direct dephosphorylation of JNK, and thus was identified as a novel negative regulator of JNK signaling pathway.

## Results

### PTPN1 is a novel JNK MAPK pathway phosphatase

**Figure 1:**
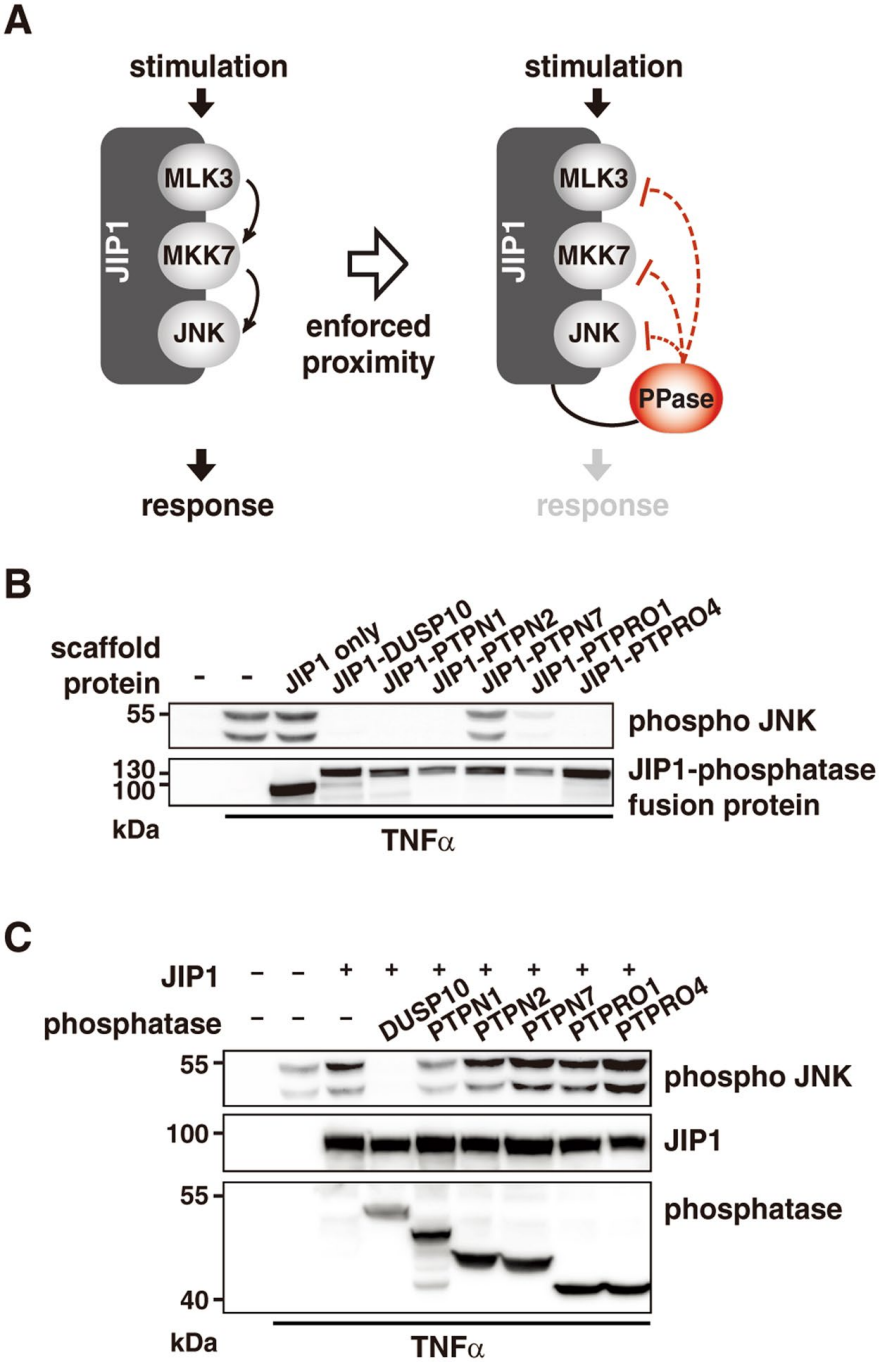
PTPN1 is a novel negative regulator of the JNK pathway. (**A)** Schematic illustration of the screening method. Phosphatases were expressed as JIP1-phosphatase fusion proteins and a decrease in JNK signaling was monitored. **(B)** The decrease of JNK phosphorylation by JIP1-phosphatase fusion was examined by immunoblot analysis using an anti-dual phospho-JNK antibody. 293 T cells were incubated for 24 h after transfection, and then treated with TNFα (15 min). **(C)** Negative regulation of the JNK pathway by the analyzed phosphatases was confirmed by co-expression of individual phosphatases and JIP1. All experiments were performed at least three times.

To focus on the specific effect of a phosphatase on JNK MAPK pathway, potential negative JNK MAPK regulators were screened using JIP1 scaffold-phosphatase fusion proteins (Fig. 1A). The effects of 77 phosphatases on pathway output were tested using various JIP1-phosphatase fusion proteins (Table S2). After TNFα-stimulation, changes in the level of JNK pathway induced by a given JIP1-phosphatase fusion protein was assessed via immunoblot analysis of phosphorylated JNK. The results showed that the level of JNK phosphorylation was decreased in case of JIP1-fused DUSP4, DUSP6, DUSP7, DUSP10, DUSP16, PTPN1, PTPN2, PTPN7, PTPRO1, and PTPRO4 (Fig. S1). Of these phosphatases, DUSP4, DUSP6, DUSP7, DUSP10, and DUSP16 have been previously reported to be negative regulators of the JNK pathway^45,46^. The effects of the remaining phosphatases (PTPN1, PTPN2, PTPN7, PTPRO1, and PTPRO4) on JNK pathway activation are unknown (Fig. 1B).

In addition, each phosphatase was individually co-expressed with JIP1 to clarify whether the observed downregulation of JNK pathway was caused by specific activity of a given phosphatase for the member of the JNK pathway, or instead nonspecifically induced by enforced proximity between the two. DUSP10 was chosen as a negative control to compare the effect of the other phosphatases on phosphorylated JNK. PTPN1 alone was found to reduce JNK phosphorylation (Fig. 1C), and furthermore, was shown to downregulate JNK signaling in both JIP1 scaffold-fused form and its free form (Fig. 1B,C).

### PTPN1 interacts with the components of the JNK MAPK pathway

**Figure 2:**
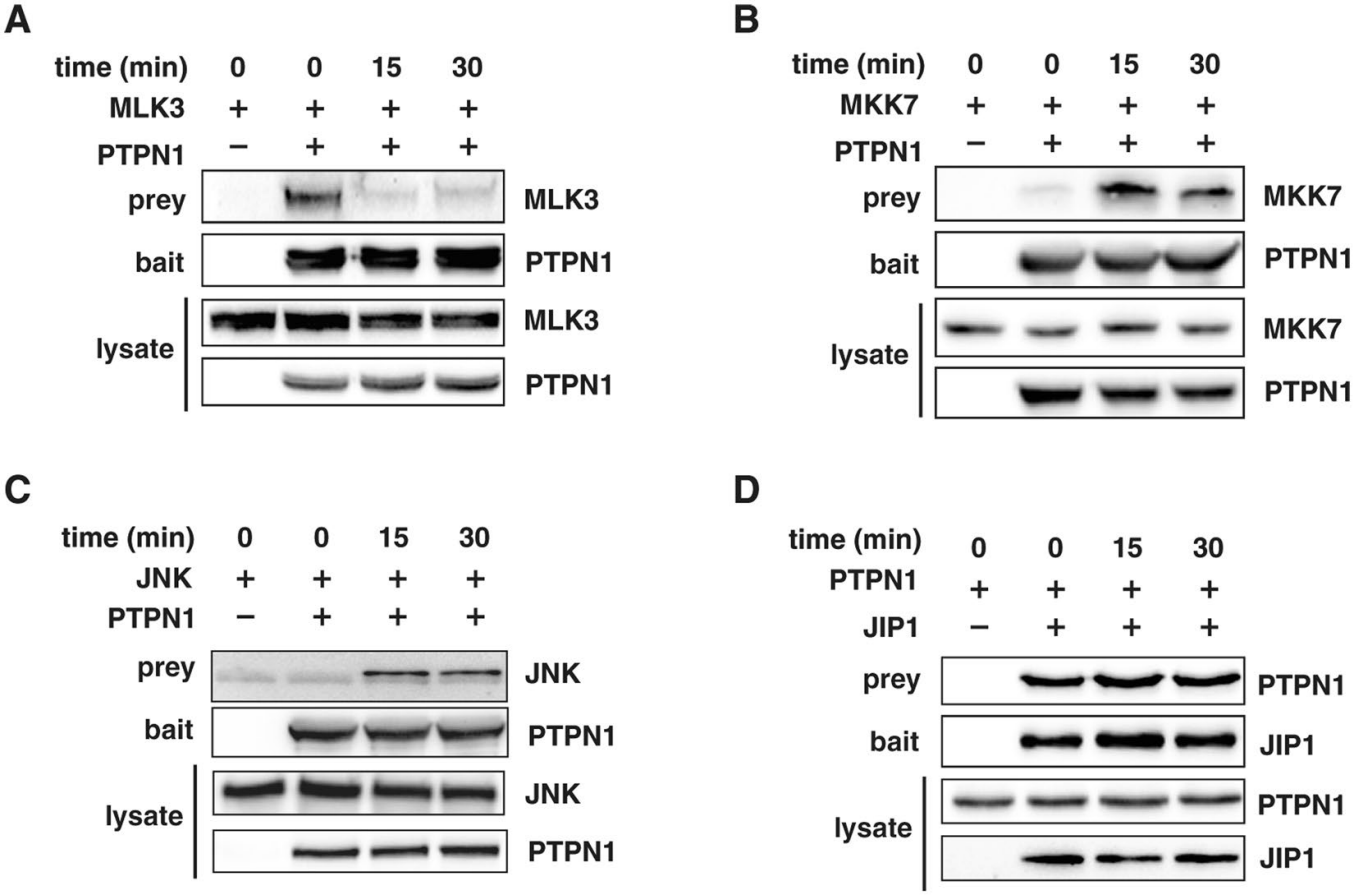
PTPN1 interacts with the components of the JNK pathway. PTPN1 interaction with **(A)** MLK3, **(B)** MKK7, **(C)** JNK, and **(D)** JIP1 was observed via immunoprecipitation assay. Each sample was prepared by TNFα-treatment at the indicated time-point. All experiments were performed at least three times.

For PTPN1 to dephosphorylate a particular JNK pathway protein, it must first interact with that protein. Thus, the interactions between PTPN1 and components of the JNK pathway in response to TNFα-stimulation were examined in 293 T cells. The results showed that PTPN1 bound to MLK3 in unstimulated cells, whereas this interaction between PTPN1 and MLK3 was decreased in TNFα-treated cells (Fig. 2A). In contrast, interaction between PTPN1 and either MKK7 or JNK was enhanced by TNFα stimulation (Fig. 2B,C), and PTPN1 interacted with the JIP1 scaffold protein independently of TNFα stimulation (Fig. 2D). A parallel *in vitro* binding analysis was performed using purified proteins and the results (Fig. S2) showed that PTPN1 can directly interact with all components of the JNK pathway including JIP1 and three kinases. JIP1 also interacted with MLK3, MKK7, and JNK regardless of TNFα stimulation in cell (Fig. S3). The fact that the pattern of interaction between JIP1 and the three kinases was notably different from that of PTPN1 and the kinases and that PTPN1 can directly bind to kinases *in vitro* suggests that the demonstrated interaction of PTPN1 with the three kinases (Fig. 2A–C) was not via its constitutive binding with the JIP1. Together, these data indicate that PTPN1 interacts with all three JNK pathway kinases, and that these interactions are not dependent upon the JIP1 scaffold.

### JNK is dephosphorylated by PTPN1

**Figure 3:**
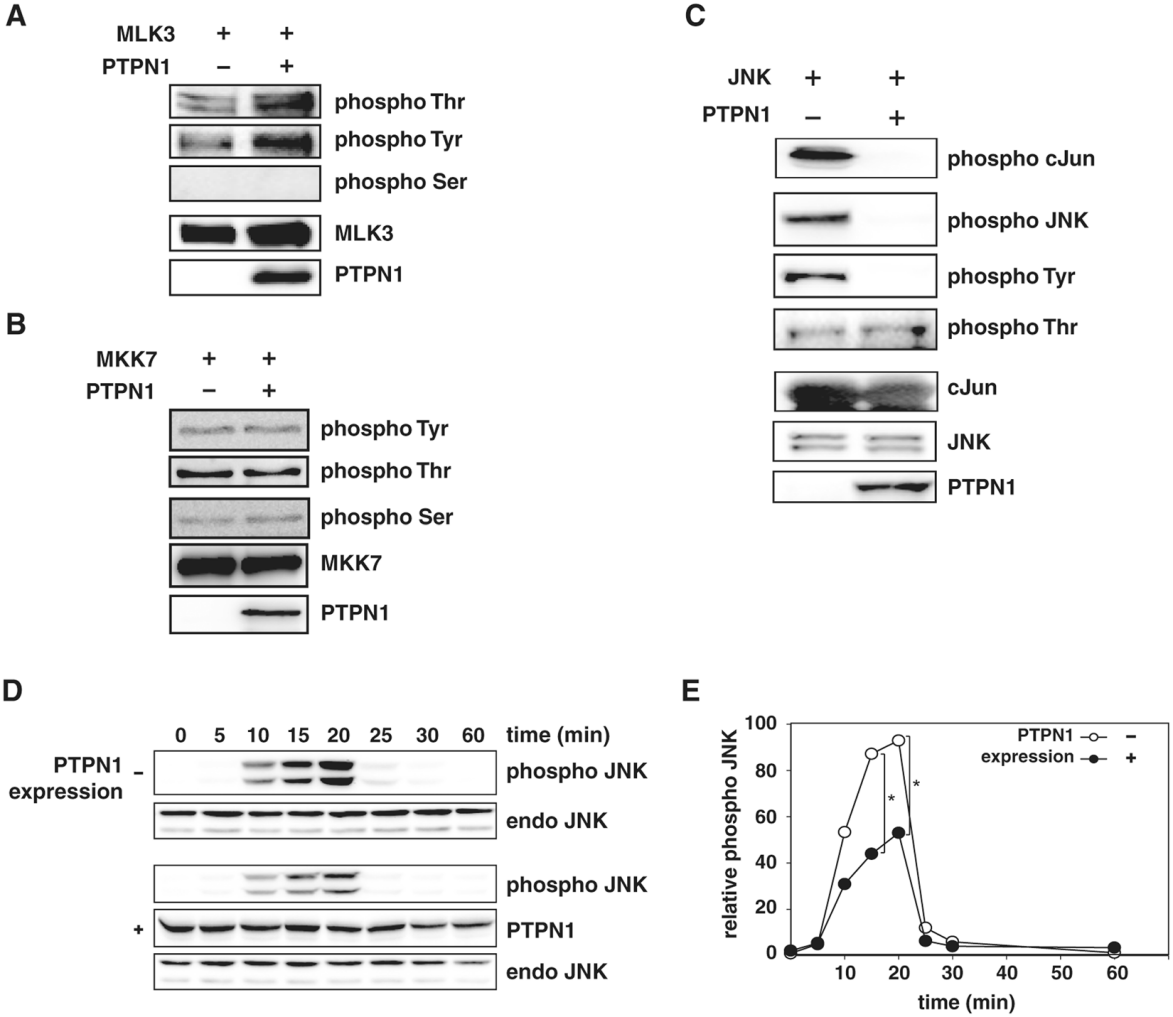
PTPN1 negatively regulates the JNK pathway via JNK dephosphorylation. (**A–C)** Phosphorylation levels of each kinase were examined by immunoblot analysis. PTPN1 was incubated with phosphorylated form of **(A)** HA-MLK3 and **(B)** GST-MKK7. **(C)** Phosphorylated GST-JNK was incubated with PTPN1 (30 min, 37 °C), and then GST-cJun and ATP were added to perform a JNK kinase assay. **(D)** Flag-PTPN1 was expressed in 293 T cells. Control cells were transfected with an empty vector. After TNFα-stimulation, samples were prepared at the indicated time-points. JNK phosphorylation levels, with or without PTPN1 expression, were examined by immunoblot analysis using an anti-dual phospho-JNK antibody. **(E)** The density of immunoblotted bands detected using the anti-dual phospho-JNK antibody is plotted. (*p < 0.05) All experiments were performed at least three times.

The JNK pathway kinases transmit signals by continuous phosphorylation of their respective downstream kinase. Thus, reduced phosphorylation of JNK by PTPN1 may be the result of inhibition of the upstream kinases, MLK3 or MKK7, or of JNK itself. A PTPN1 phosphatase assay was performed *in vitro* to ascertain whether MLK3, MKK7, or JNK might be PTPN1 substrates. When phosphorylated MLK3 or MKK7 was incubated with PTPN1, there was no significant change in their phosphorylation status (Fig. 3A,B). Surprisingly, PTPN1 was found to reduce the tyrosine phosphorylation of JNK but the level of phosphothreonine did not change (Fig. 3C). A JNK kinase assay was also performed to investigate the effect of reduced tyrosine phosphorylation on JNK activity. After incubation of phosphorylated JNK with PTPN1, JNK failed to phosphorylate GST-cJun (Fig. 3C). This suggests that JNK is a novel PTPN1 substrate, and that PTPN1 reduces the catalytic activity of JNK by reducing tyrosine phosphorylation of JNK *in vitro*.

To elucidate the mechanism by which PTPN1 regulates the overall JNK signaling, we examined the level of phosphorylated JNK at regular intervals after treatment with TNFα (Fig. 3D,E). When PTPN1 was expressed, JNK phosphorylation was reduced by approximately half compared to that achieved in the absence of PTPN1 expression. However, the timing of JNK activation or deactivation was not affected by PTPN1 expression.

### PTPN1 negatively regulates the JNK MAPK pathway and inhibits the cell death response

**Figure 4:**
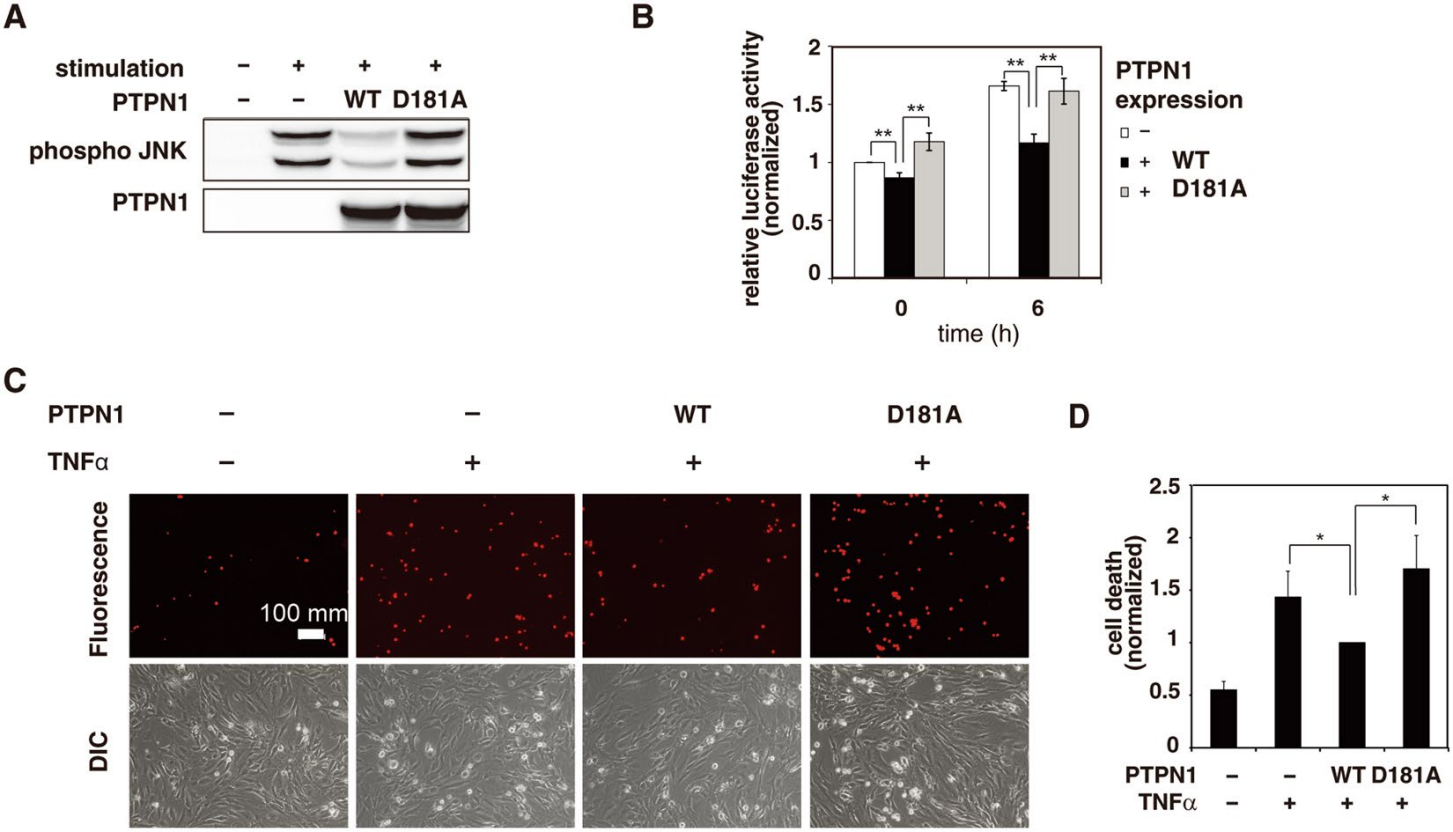
PTPN1 regulates JNK-related cell death response. (**A)** Flag-PTPN1 or Flag-PTPN1 D181A was expressed in 293 T cells. Control cells were transfected with an empty vector. Upon TNFα-stimulation, protein expression and JNK phosphorylation were each examined by immunoblotting. **(B)** JNK activity was assessed using a luciferase reporter gene assay. The data are presented as the mean ± SD (repeats were performed in triplicate). (**p < 0.005) **(C)** Flag-PTPN1 or FLANG-PTPN1 D181A was expressed in p65-deficient MEF cells. After TNFα-stimulation (7 h), dead cells were visualized with PI staining (7 µg/ml) and observed via microscopy. **(D)** The relative cell death ratio was plotted ( ± SD, n = 3). (*p < 0.05).

When 293 T cells were treated with TNFα, those expressing PTPN1 were shown to exhibit reduced levels of JNK phosphorylation (Fig. 3). To further evaluate the function of PTPN1, experiments using a catalytic PTPN1 mutant (PTPN1 D181A) were performed. Data showed that PTPN1 D181A bound to JNK as efficiently as wild-type (WT) PTPN1 did, but exhibited reduced catalytic activity against JNK (Figs S4 and S5). Resultantly, although the binding affinity of PTPN1 D181A for JNK was similar to that of WT PTPN1, its expression did not reduce the level of JNK phosphorylation as WT PTPN1 did (Fig. 4A). This observation was also confirmed by a luciferase reporter gene assay to monitor JNK catalytic activity (Fig. 4B). Together, WT PTPN1 decreased both JNK phosphorylation and luciferase activity, while PTPN1 D181A increased luciferase activity via prolonged JNK phosphorylation (Fig. 4A,B). These results suggest that PTPN1 promotes JNK pathway inactivation.

It is known that the JNK pathway is transiently activated when cells are stimulated with TNFα, because the NFκB pathway induces expression of MnSOD and thereby prevents not only the accumulation of ROS, but also the oxidation and inactivation of PTPs by ROS^47,48^. Thus, it is difficult to observe the cell death response caused by JNK pathway in 293 T cells that harbor NFκB pathway. Since the sustained JNK activation is necessary for TNFα-induced apoptosis, the cell death response was examined in p65-deficient MEF cells, in which the NFκB pathway was disrupted^2,14^. The results showed that PTPN1 decreased TNFα-stimulated cell death responses, whereas PTPN1 D181A increased the cell death response presumably via sustained JNK phosphorylation (Fig. 4C,D). These results demonstrate that PTPN1 reduces the cell death response via negative regulation of the JNK pathway.

## Discussion

The formation of protein complexes often mediated by scaffold proteins increases the proximity between signaling components, and thereby facilitates signal transduction^7,8,49^. Scaffold protein-mediated MAPK pathway signals are transmitted by relayed phosphorylation of member kinases^3,4^. Malfunction of the signaling components or dysregulation of the signal transduction pathway have been previously investigated as putative therapeutic targets^15^. In this study, identification of a negative regulator of the JNK pathway was achieved via a facile synthetic screening approach using scaffold protein-tethered phosphatase fusion proteins (Fig. 1B). This was possible because when JIP1 interacts with JNK pathway kinases during the signal transduction process, a phosphatase expressed as a scaffold-fused protein is more accessible to kinases than a free form of phosphatase is. Furthermore, the effect of each phosphatase increased as a result of close proximity to the JIP1 signaling complex, and this augmented effect enabled the identification of PTPN1 as a novel negative JNK pathway regulator. PTPN1 was shown to catalyze JNK inactivation by dephosphorylating tyrosine residue in an active JNK (Fig. 3C). The utilized anti-dual phospho-JNK antibody can capture phosphorylated JNK only when both the threonine and tyrosine residues of the TPY motif in the activation loop of JNK are phosphorylated to produce a fully activated form of JNK^50^. Therefore, the decrease in the dual phospho-JNK and phospho-Tyr blottings by PTPN1 (Fig. 3C) suggests that PTPN1 may directly dephosphorylate the phosphotyrosine residue present in the TPY motif.

In eukaryotic cells, external cues trigger the onset of MAPK signaling and activate intracellular signaling components to generate corresponding cellular responses, and the signaling is eventually decreased to its basal level after certain amount of time, which is often called adaptation^51^. Precise control of the magnitude and duration of signaling is critical for cell survival. Dysregulation of these mechanisms usually leads to disease states as shown in tumorous diseases where adaptation of proliferation signaling is malfunctioning and uncontrolled proliferation is manifested^14,16^. Adaptation of signaling is often mediated by degradation of active signaling proteins or negative feedback regulations involving protein phosphatases. Dephosphorylation of active signaling proteins by phosphatases is a particularly important mechanism in the JNK MAPK pathway where the main signaling components are kinases. In our study, the expression of PTPN1 decreased the overall signaling magnitude, but not the duration of JNK signaling (Fig. 3D,E), suggesting that its primary function appears to control “signaling noise” during signaling process, rather than regulating the duration of JNK activation or the signal adaptation.

We also showed the catalytic mutant PTPN1 D181A could bind to JNK as efficiently as WT PTPN1 did. PTPN1 D181A failed to inhibit TNFα-stimulated JNK phosphorylation, and led to an increased cell death in p65-deficient MEF cells (Fig. 4). These results indicated that the negative regulation of TNFα-dependent JNK signaling by PTPN1 depends on its catalytic activity. Thus, elucidation of the functional mechanism of PTPN1 may help to understand the JNK-dependent cell death, which is a prominent feature in several diseases, such as cancer, diabetes, inflammatory diseases, and neurodegenerative diseases^14,16^.

Interestingly, PTPN1 directly binds to JIP1 scaffold and three kinases of JNK MAPK pathway *in vitro* (Fig. S2), while exhibiting dynamic changes in its interactions with JNK pathway components in response to TNFα stimulation (Fig. 2). This finding suggests a possibility of an additional non-catalytic role of PTPN1 in the regulation of JNK signaling, for example, sequestering of pathway members in the resting cells or acting as a co-scaffold. Previous studies have revealed non-catalytic functions of various phosphatases in the signal regulation^47,48,52,53^. PTPN1 has been well established for its regulatory role in insulin signaling and inflammatory signaling which involves JNK and p38 pathways in the downstream^37,54,55^. JNK MAPK pathway is often integrated with p38 MAPK pathway since they share multiple upstream components and stimuli which activate the both^56,57^, suggesting that PTPN1 may potentially regulate the phosphorylation of p38^58^. Thus, studying the functional mechanisms and physiological roles of PTPN1 in JNK MAPK pathway should contribute to a broader understanding of the regulation and integration of MAPK signaling.

## Methods

### Cell lines and transfection

HEK293 T (293 T) and MEF cells were cultured in Dulbecco Modified Eagle Medium (DMEM; Gibco) supplemented with 10% (v/v) FBS (Gibco)^59,60^. 293 T cells were transfected with appropriate plasmids using D-fection (LugenSci) according to manufacturer’s instructions. MEF cells were transfected by electroporation using the Neon transfection system (Invitrogen). Prior to treatment with TNFα, cells were incubated in serum-free medium (2 h), and then stimulated with TNFα (BD) for the indicated time.

### Plasmids for protein expression

The plasmid constructs used for protein expression are listed (Table S1). The JIP1 gene was cloned from a mouse brain cDNA library using PCR^61^. To construct the JIP1 scaffold-tethered phosphatase proteins, phosphatases were fused to the C-terminus of JIP1 with a linker composed of Gly and Ser residues (Ser-Gly-Gly-Gly-Ser). The phosphatases used in this study are listed (Table S2). PTPN1 D181A was generated by introducing point mutations to PTPN1 using PCR mutagenesis.

### Immunoblotting and immunoprecipitation

Immunoblotting and immunoprecipitation were performed as previously described^61^. The antibodies used in this study were: anti-dual phospho-JNK (Cell Signaling), anti-Flag (Sigma), anti-HA (Boehringer Mannheim), anti-phospho-Thr (Upstate), anti-phospho-Tyr (Upstate), anti-phospho-Ser (Upstate), anti-His (Sigma), anti-GST (GE), anti-JIP1 (Santa Cruz), and anti-myc (Upstate). In all cases, before performing gel-electrophoresis, the amount of total proteins was quantified using Bradford assay and equal amounts of proteins were loaded in each lane in a gel. All immunoblot analyses were independently performed at least three times.

### Kinase assay using immunoblotting

To test JNK catalytic activity, protein kinase activity was assayed using GST-cJun as a substrate. Samples were incubated (30 min) with GST-cJun in a kinase assay buffer (25 mM HEPES pH 7.0, 20 mM MgCl_2_, 2 mM DTT, and 20 μM ATP), and then examined via immunoblot analysis using an anti-dual phospho-cJun antibody (Cell Signaling).

### Cell death assay

p65-deficient MEF cells were transfected with either p3xFLAG CMV-PTPN1 WT, PTPN1 D181A, or empty vector, and incubated for 24 h. To stimulate the cell death response, cells were treated with TNFα (50 nM) for 7 h after undergoing serum starvation. Dead cells were visualized with PI staining (7 μg/ml) in DMEM, and detected using a DE/Axiovert 200 inverted microscope (Carl Zeiss). Cells were counted from DIC and fluorescence images in triplicate, to calculate cell death percentages.

### Pull down assay

An *in vitro* binding assay was performed with bacterially expressed proteins. Prey and bait proteins were incubated (2 h), and glutathione sepharose (GE) was incubated with protein samples (2 h). Precipitates were washed (five times) with PBS, and then examined by immunoblot analysis.

### Phosphatase assay

The catalytic activity of both PTPN1 and PTPN1 D181A was measured using pNPP. To identify PTPN1 substrates, phosphatase assays were performed with bacterially expressed PTPN1. HA-MLK3 was expressed in 293 T cells, and then purified using anti HA-agarose (Sigma). To purify phospho-MKK7, GST-MKK7 was co-expressed with His-MLK3 in *Escherichia coli*. To generate phosphorylated JNK, GST-JNK was co-expressed with His-MKK7, (a constitutively activated mutant form of MKK7, harboring Ser-271, Thr-275, and Ser-277 mutations to Glu) in *Escherichia coli*. Phosphorylated proteins were incubated with purified His-PTPN1 (30 min, 37 °C). Protein phosphorylation levels were detected by immunoblot analysis.

### Reporter gene assay

293 T cells were transfected with pFA2-cJun, pFR-Luc, and p3xFLAG CMV-PTPN1 (WT, PTPN1 D181A, or empty vector), and incubated for 24 h. After serum starvation (2 h), cells were treated with TNFα for the indicated time periods. Samples were prepared for, and analyzed by luciferase assay (Promega), according to the manufacturer’s instructions.

### Statistical analysis

Values were expressed as means ± SD. One-way ANOVA and Dunnett’s t-test was used for multiple comparisons using GraphPad Prism (GraphPad Software, La Jolla, CA, USA). The criteria for statistical significance were set at *p < 0.05 and **p < 0.005.

### Data Availability

No datasets were generated or analyzed during the current study.

## Electronic supplementary material

Supplementary Information
