# Supplementary material for: Identification of PTPN1 as a novel negative regulator of the JNK MAPK pathway using a synthetic screening for pathway-specific phosphatases
Source: Sci Rep. 2017 Oct 11;7:12974. doi: 10.1038/s41598-017-13494-x (PMC5636874; doi:10.1038/s41598-017-13494-x)
Supplement: Supplementary file 1 — Supplementary Information [file 41598_2017_13494_MOESM1_ESM.doc]

Supplementary Information for

**Identification of PTPN1 as a novel negative regulator of the JNK MAPK pathway using a synthetic screening for pathway-specific phosphatases**

Jiyoung Moon ^a, †^, Jain Ha ^a, †^, and Sang-Hyun Park ^a,*^

^a^ Department of Biological Sciences, Seoul National University, Seoul 08826, Korea


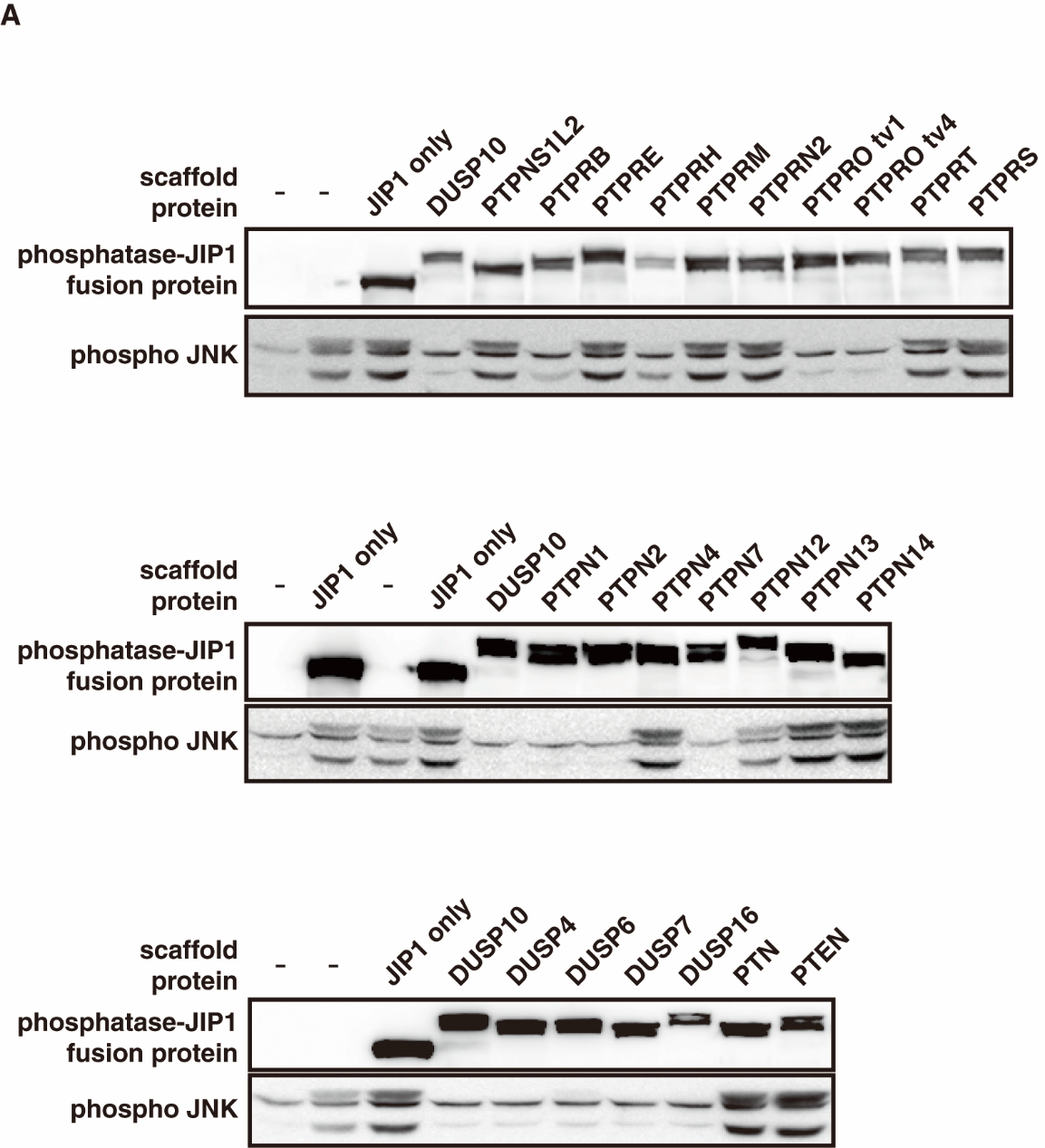


**
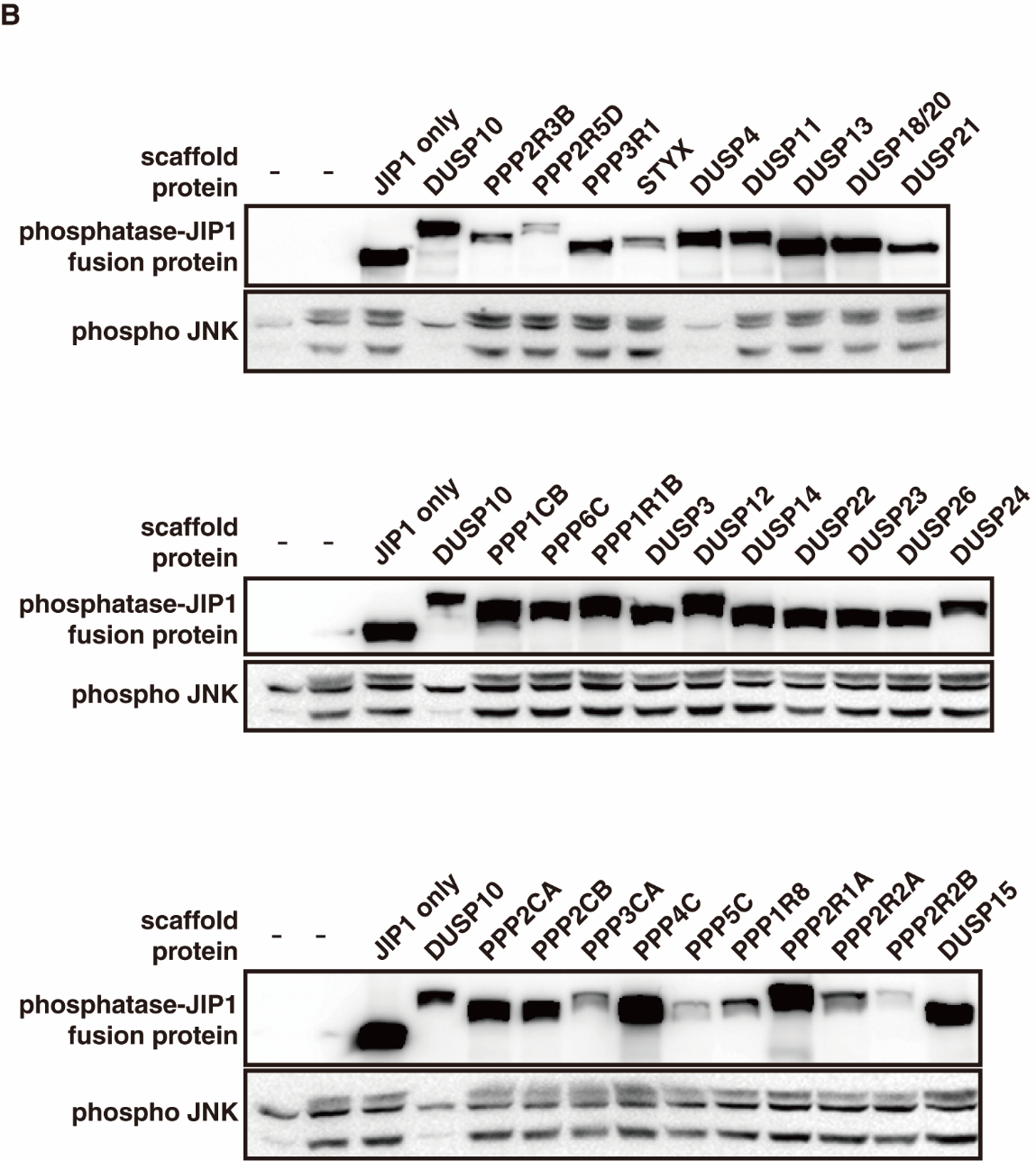
**

**
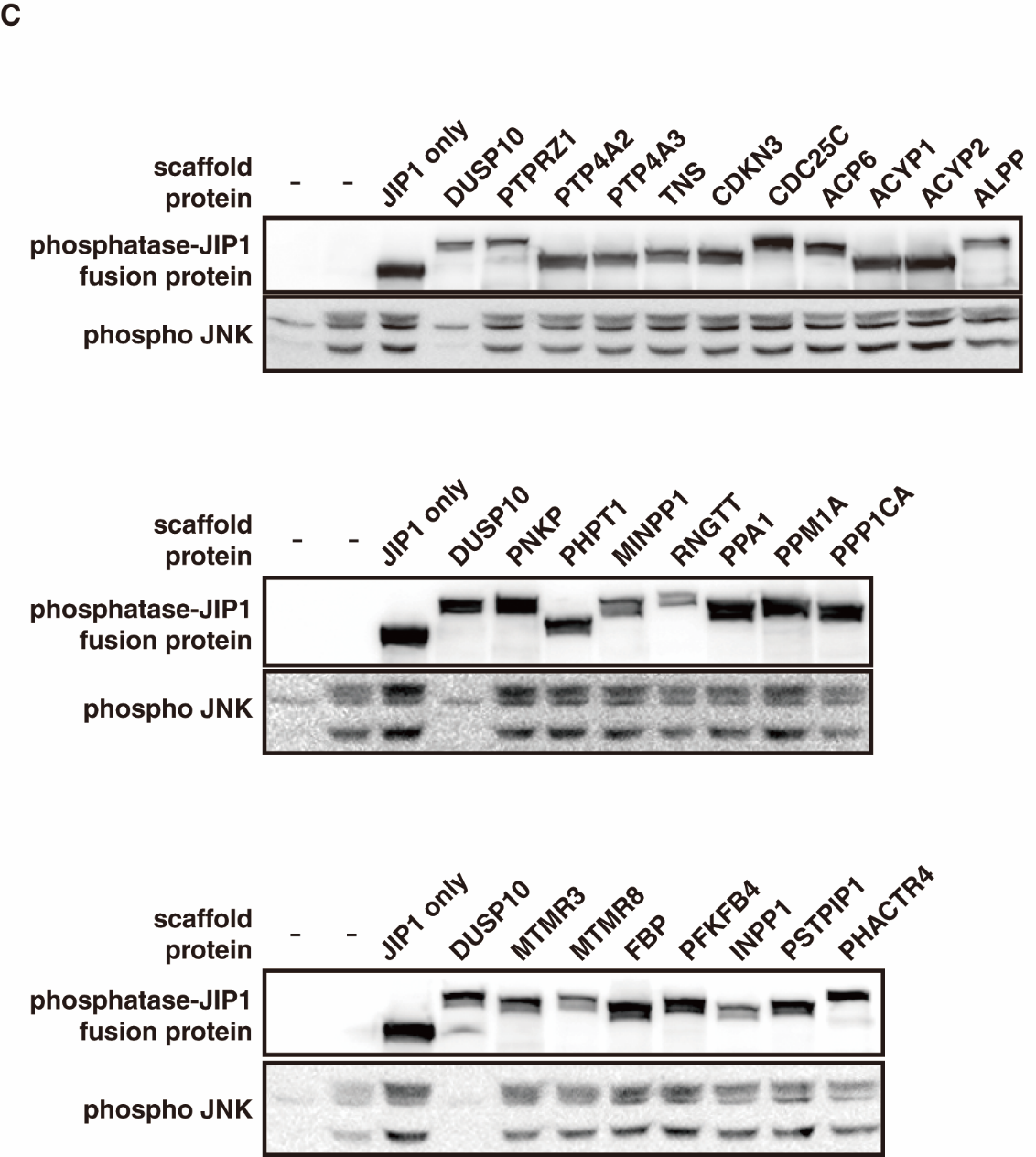
**

**Figure S1. In order to find novel regulators of JNK pathway, 77 phosphatases were examined by the screening using JIP1-phosphatase fusion protein. (A)** protein tyrosine phosphatases, **(B)** dual specificity phosphatases, **(C)** protein Ser/Thr phosphatases and other phosphatases were expressed as JIP1-phosphatase fusions in 293T cells. After transfection (24 h), cells were treated with TNFα (15 min). Reduced activation of JNK pathway was examined by immunoblot analysis using an anti-dual phospho-JNK antibody. All experiments were performed at least three times.


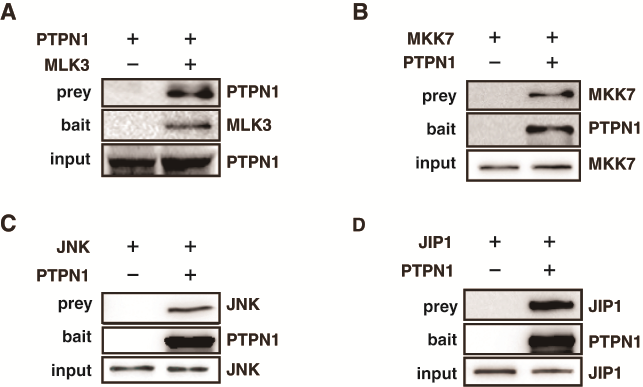


## **Figure S2. PTPN1 directly binds to all the components of JNK pathway. (A-D)**. Pull-down assay with glutathione sepharose were performed using bacterially expressed proteins. Bindings of PTPN1 with **(A)** MLK3, **(B)** MKK7, **(C)** JNK and **(D)** JIP1 were examined by immunoblot analysis. All experiments were performed at least three times.


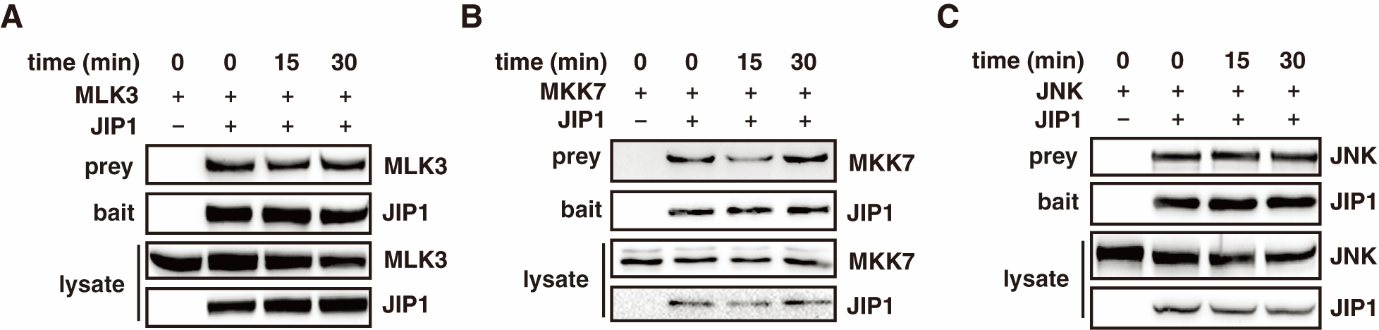


**Figure S3. Bindings of PTPN1 to components in response to TNFα were not mediated by JIP1. (A-C)**. Flag-JIP1 was over-expressed together with each component of the JNK pathway, including **(A)** MLK3, **(B)** MKK7, and **(C)** JNK in 293T cells. Cells were treated with TNFα, and then samples were prepared at the indicated time-points. Interactions of JIP1 with components were examined by immunoprecipitation assay and immunoblot analysis. All experiments were performed at least three times.


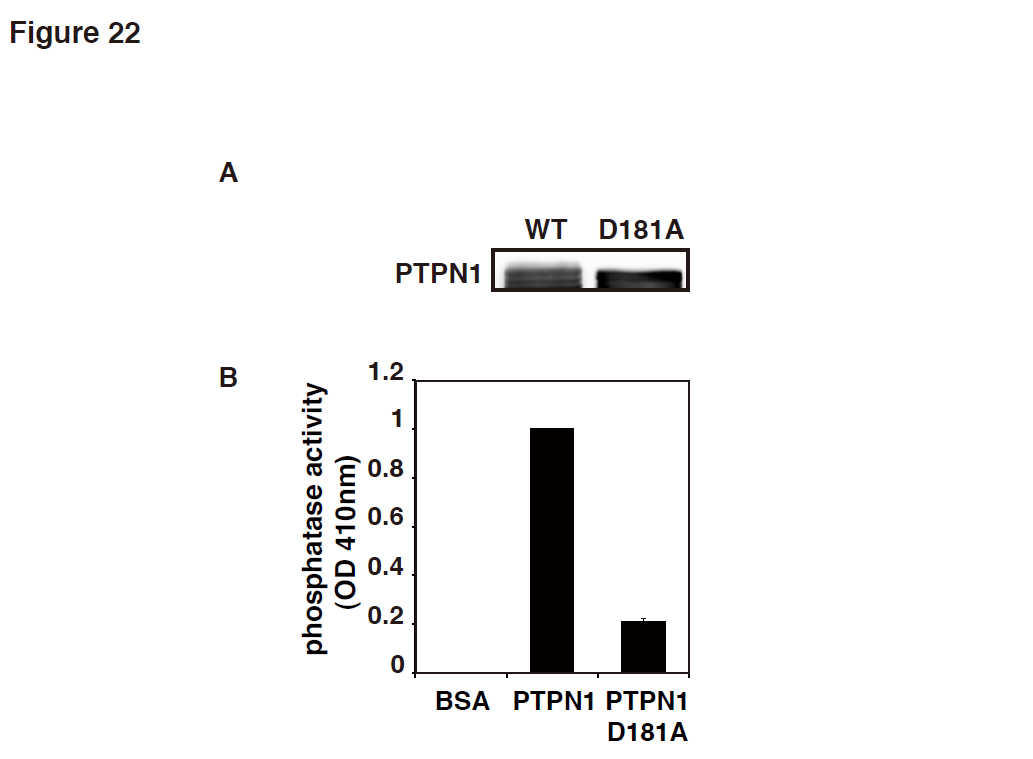


**Figure S4. Activity of WT PTPN1 and PTPN1 D181A was measured by pNPP assay. (A)** His-tagged WT PTPN1 and inactive mutants, PTPN1 D181A, were expressed in BL21 Rosetta cells and then purified by using Ni sepharose. Purified phosphatases were examined by immunoblot analysis using anti-His antibody. **(B)** Activity of these phosphatases was tested by pNPP assay. The relative ratio of phosphatase activity is plotted. The data in the bar graphs are the mean ±SD of triplicate experiments.


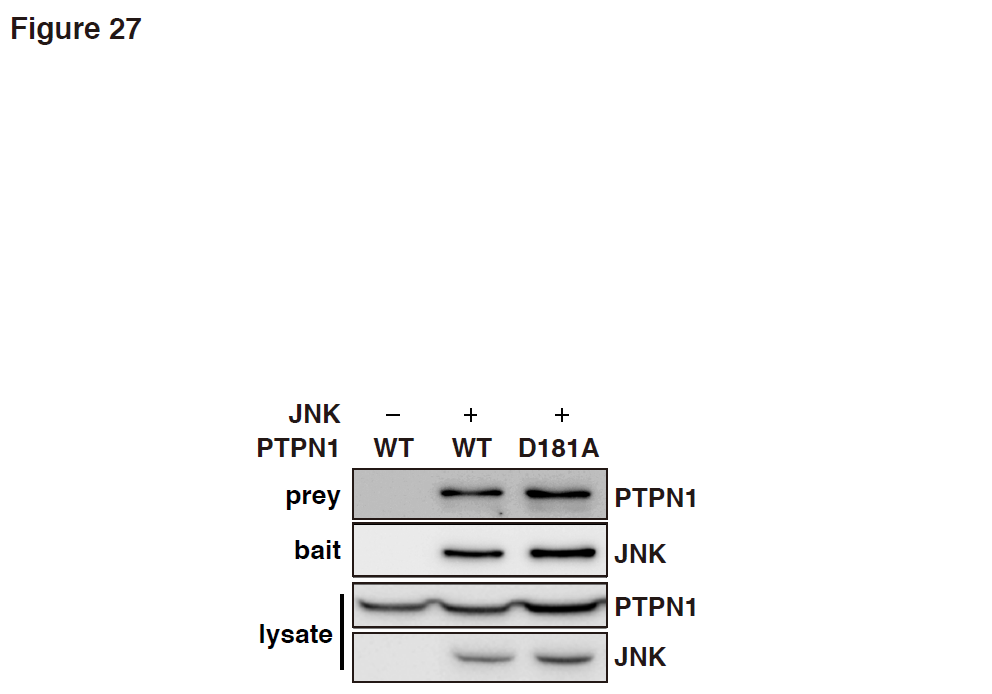


**Figure S5. PTPN1 D181A and WT bind to JNK with similar affinity.** Flag-PTPN1 or Flag-PTPN1 D181A was expressed with JNK-myc in 293T cells. Binding of JNK was examined by immunoprecipitation assay using anti-myc antibody-conjugated agarose and immunoblot analysis. These results were replicated at least three times.

**
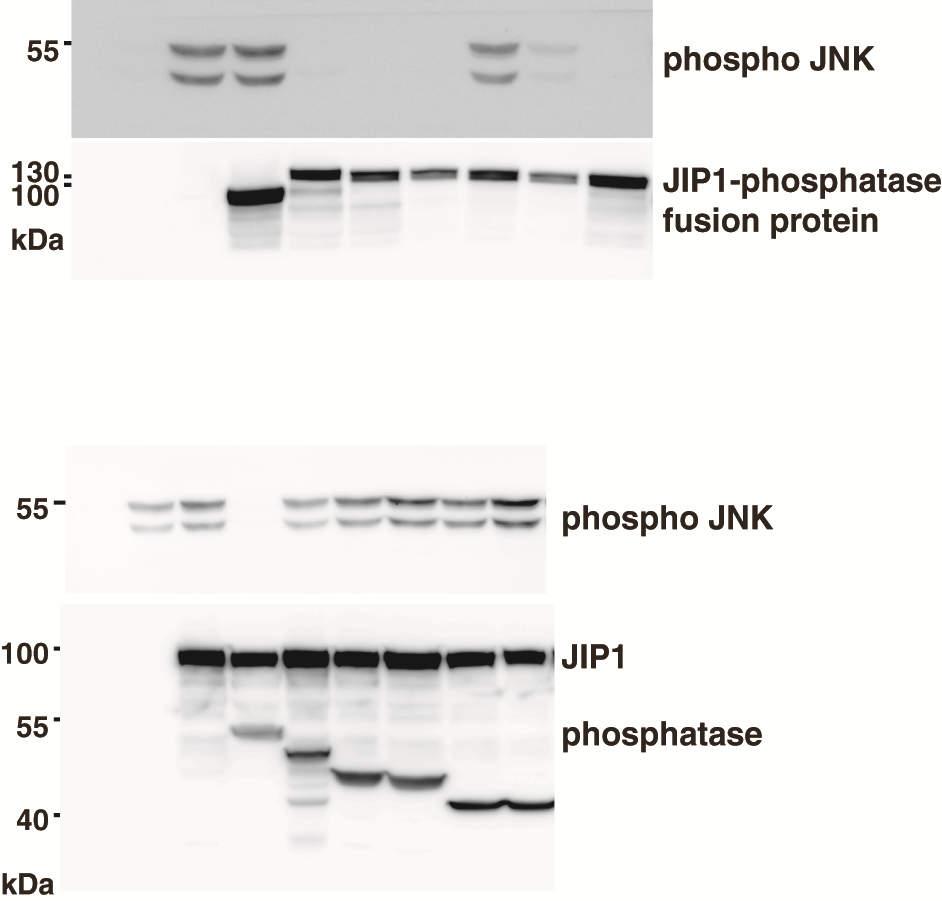
**

**Figure S6. Full-length blots displayed in Fig. 1B (top) and 1C (bottom).**


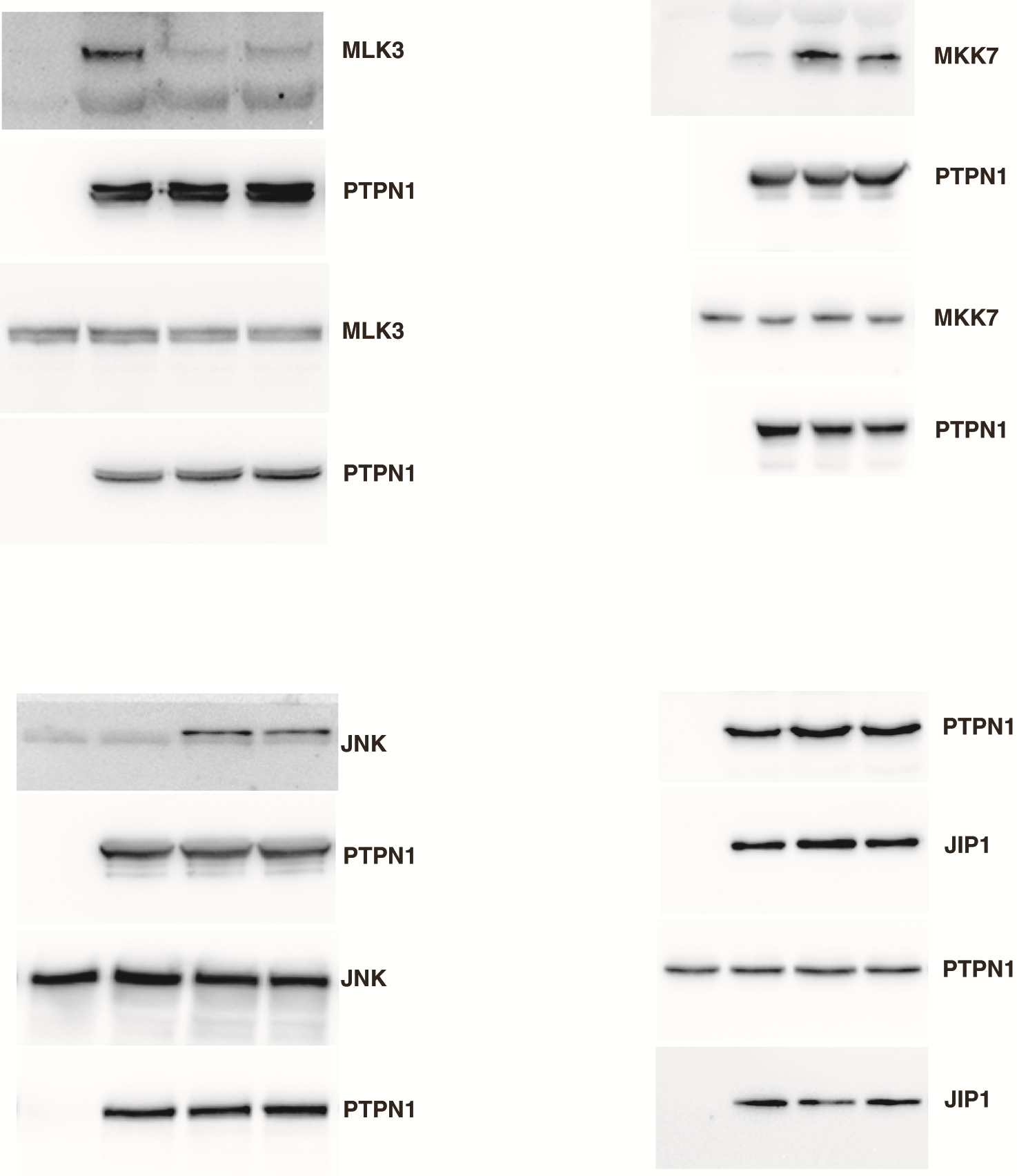


**Figure S7. Full-length blots displayed in Fig. 2A (top left), 2B (top right), 2C (bottom left) and 2D (bottom right).**

**
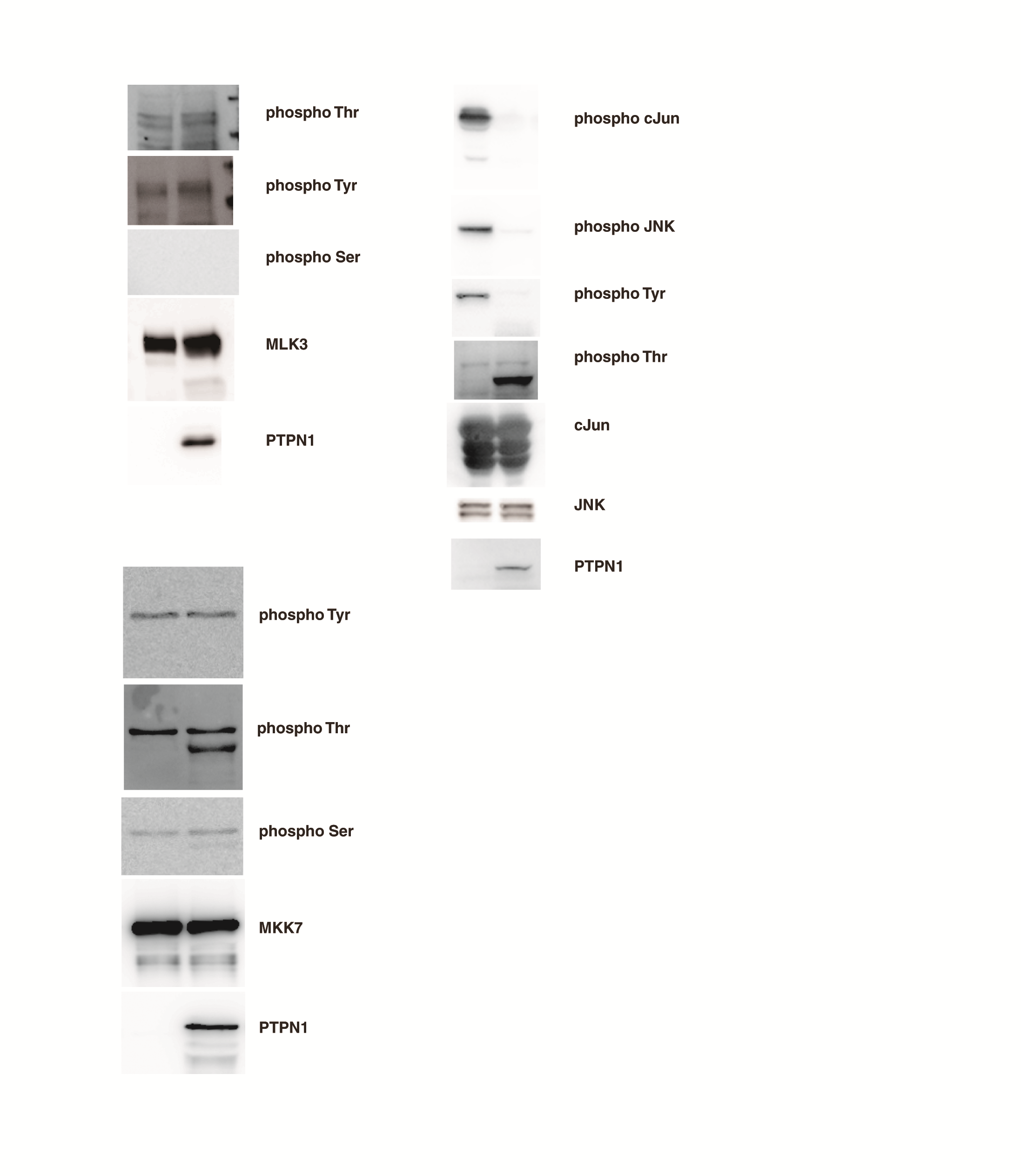
**

**Figure S8. Full-length blots displayed in Fig. 3A (top left), 3B (bottom left) and 3C (right).**

**
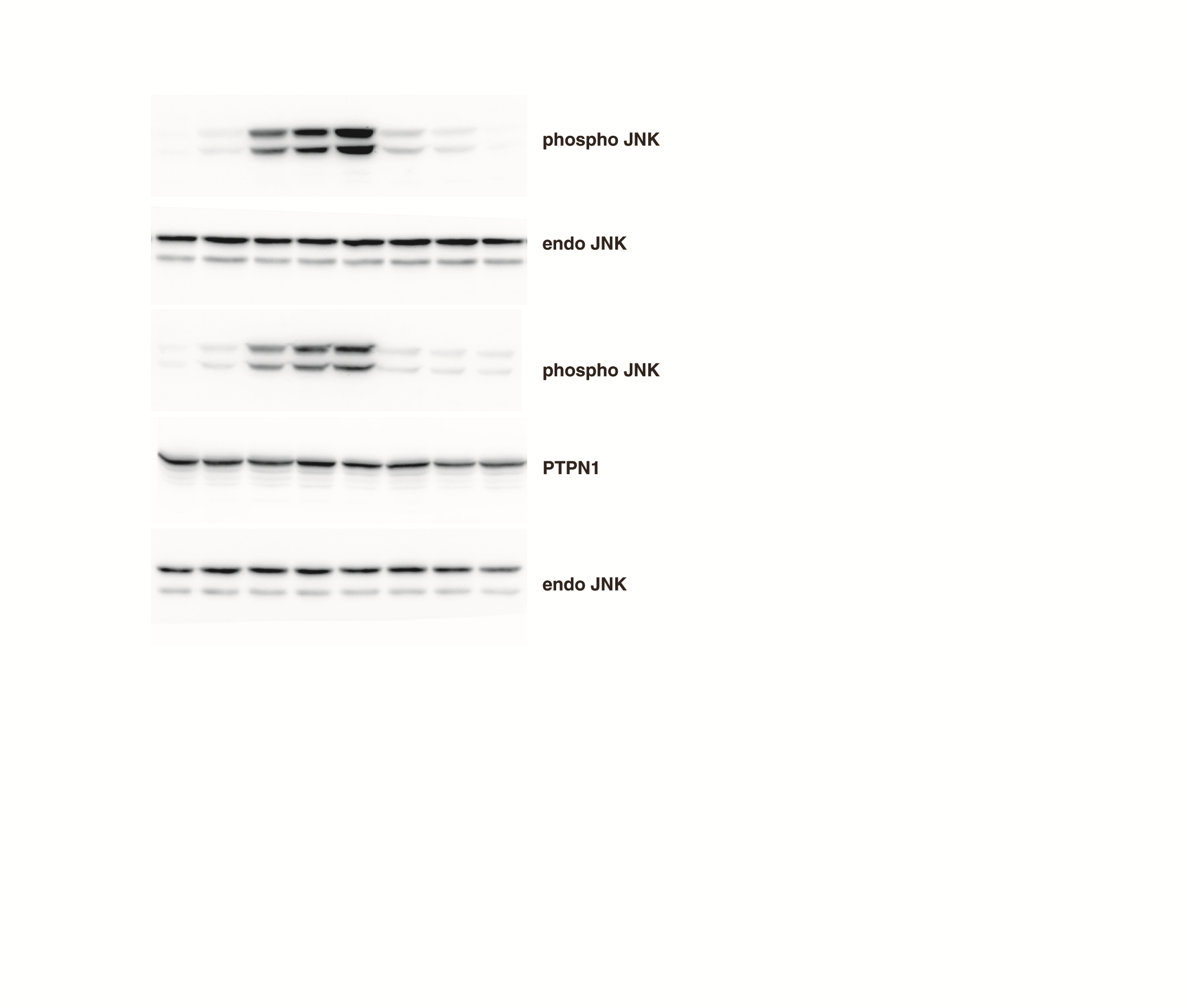
**

**Figure S9. Full-length blots displayed in Fig. 3D.**

**
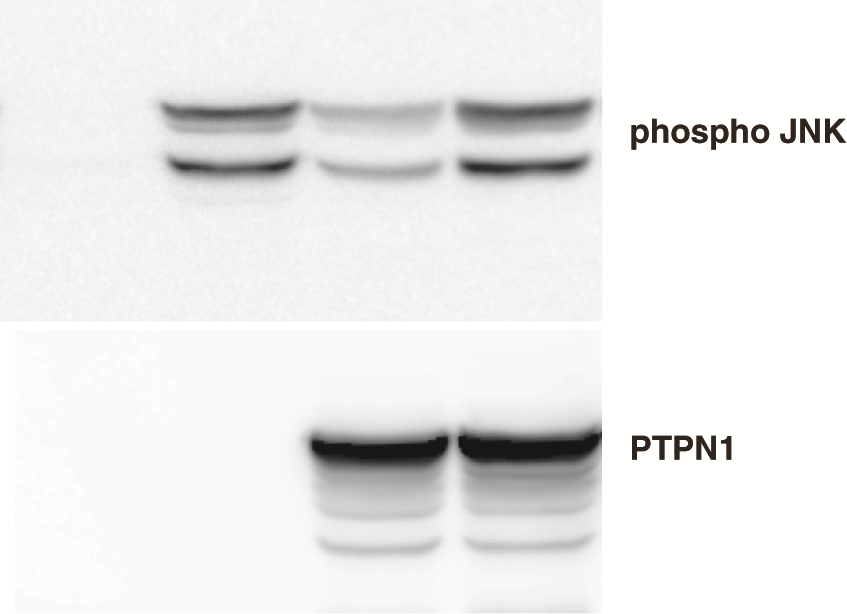
**

**Figure S10. Full-length blots displayed in Fig. 4A.**

**Table S1. Plasmid constructs used in this study were listed.**

|  |  | |
| --- | --- | --- |
| Screening for novel regulators of JNK MAP kinase pathway | | |
| names | | descriptions |
| pSH2732 | | Flag-JIP1 in p3xFLAG-CMV |
| pSH2733 | | Flag-JIP1-DUSP3 in p3xFLAG-CMV |
| pSH2734 | | Flag-JIP1-DUSP4 in p3xFLAG-CMV |
| pSH2735 | | Flag-JIP1-DUSP6 in p3xFLAG-CMV |
| pSH2736 | | Flag-JIP1-DUSP7 in p3xFLAG-CMV |
| pSH2737 | | Flag-JIP1-DUSP10 in p3xFLAG-CMV |
| pSH2738 | | Flag-JIP1-DUSP11 in p3xFLAG-CMV |
| pSH2739 | | Flag-JIP1-DUSP12 in p3xFLAG-CMV |
| pSH2740 | | Flag-JIP1-DUSP13 in p3xFLAG-CMV |
| pSH2741 | | Flag-JIP1-DUSP14 in p3xFLAG-CMV |
| pSH2742 | | Flag-JIP1-DUSP15 in p3xFLAG-CMV |
| pSH2743 | | Flag-JIP1-DUSP16 in p3xFLAG-CMV |
| pSH2744 | | Flag-JIP1-DUSP18/20 in p3xFLAG-CMV |
| pSH2746 | | Flag-JIP1-DUSP22 in p3xFLAG-CMV |
| pSH2747 | | Flag-JIP1-DUSP23 in p3xFLAG-CMV |
| pSH2748 | | Flag-JIP1-DUSP26 in p3xFLAG-CMV |
| pSH2749 | | Flag-JIP1-MK-STYX in p3xFLAG-CMV |
| pSH2750 | | Flag-JIP1-PPA1 in p3xFLAG-CMV |
| pSH2751 | | Flag-JIP1-PPM1A in p3xFLAG-CMV |
| pSH2752 | | Flag-JIP1-PPP1CA in p3xFLAG-CMV |
| pSH2753 | | Flag-JIP1-PPP1CB in p3xFLAG-CMV |
| pSH2754 | | Flag-JIP1-PPP2CA in p3xFLAG-CMV |
| pSH2755 | | Flag-JIP1-PPP2CB in p3xFLAG-CMV |
| pSH2756 | | Flag-JIP1-PPP3CA in p3xFLAG-CMV |
| pSH2757 | | Flag-JIP1-PPP4C in p3xFLAG-CMV |
| pSH2758 | | Flag-JIP1-PPP5C in p3xFLAG-CMV |
| pSH2759 | | Flag-JIP1-PPP6C in p3xFLAG-CMV |
| pSH2760 | | Flag-JIP1-PPP1R1B in p3xFLAG-CMV |
| pSH2761 | | Flag-JIP1-PPP1R8 in p3xFLAG-CMV |
| pSH2745 | | Flag-JIP1-DUSP21 in p3xFLAG-CMV |

| names | descriptions |
| --- | --- |
| pSH2762 | Flag-JIP1-PPP2R1A in p3xFLAG-CMV |
| pSH2763 | Flag-JIP1-PPP2R2A in p3xFLAG-CMV |
| pSH2764 | Flag-JIP1-PPP2R2B in p3xFLAG-CMV |
| pSH2765 | Flag-JIP1-PPP2R3B in p3xFLAG-CMV |
| pSH2766 | Flag-JIP1-PPP2R5D in p3xFLAG-CMV |
| pSH2767 | Flag-JIP1-PPP3R1 in p3xFLAG-CMV |
| pSH2768 | Flag-JIP1-PTPN1 in p3xFLAG-CMV |
| pSH2769 | Flag-JIP1-PTPN2 in p3xFLAG-CMV |
| pSH2770 | Flag-JIP1-PTPN4 in p3xFLAG-CMV |
| pSH2771 | Flag-JIP1-PTPN6 in p3xFLAG-CMV |
| pSH2772 | Flag-JIP1-PTPN7 in p3xFLAG-CMV |
| pSH2773 | Flag-JIP1-PTPN12 in p3xFLAG-CMV |
| pSH2774 | Flag-JIP1-PTPN13 in p3xFLAG-CMV |
| pSH2775 | Flag-JIP1-PTPN14 in p3xFLAG-CMV |
| pSH2776 | Flag-JIP1-PTPNS1L2 in p3xFLAG-CMV |
| pSH2777 | Flag-JIP1-PTPRB in p3xFLAG-CMV |
| pSH2778 | Flag-JIP1-PTPRE in p3xFLAG-CMV |
| pSH2779 | Flag-JIP1-PTPRH in p3xFLAG-CMV |
| pSH2780 | Flag-JIP1-PTPRM in p3xFLAG-CMV |
| pSH2781 | Flag-JIP1-PTPRN2 in p3xFLAG-CMV |
| pSH2782 | Flag-JIP1-PTPRO(transcript variant 1) in p3xFLAG-CMV |
| pSH2783 | Flag-JIP1-PTPRO(transcript variant 4) in p3xFLAG-CMV |
| pSH2784 | Flag-JIP1-PTPRT in p3xFLAG-CMV |
| pSH2785 | Flag-JIP1-PTPRS in p3xFLAG-CMV |
| pSH2786 | Flag-JIP1-PTPRZ1 in p3xFLAG-CMV |
| pSH2787 | Flag-JIP1-PTP4A2 in p3xFLAG-CMV |
| pSH2788 | Flag-JIP1-PTP4A3 in p3xFLAG-CMV |
| pSH2789 | Flag-JIP1-PTEN in p3xFLAG-CMV |
| pSH2790 | Flag-JIP1-tensin in p3xFLAG-CMV |

| names | descriptions |
| --- | --- |
| pSH2796 | Flag-JIP1-ACYP2 in p3xFLAG-CMV |
| pSH2791 | Flag-JIP1-CDKN3 in p3xFLAG-CMV |
| pSH2792 | Flag-JIP1-CDC25C in p3xFLAG-CMV |
| pSH2793 | Flag-JIP1-STNS in p3xFLAG-CMV |
| pSH2794 | Flag-JIP1-ACP6 in p3xFLAG-CMV |
| pSH2795 | Flag-JIP1-ACYP1 in p3xFLAG-CMV |
| pSH2797 | Flag-JIP1-ALPP in p3xFLAG-CMV |
| pSH2798 | Flag-JIP1-MTMR3 in p3xFLAG-CMV |
| pSH2799 | Flag-JIP1-MTMR8 in p3xFLAG-CMV |
| pSH2800 | Flag-JIP1-FBP in p3xFLAG-CMV |
| pSH2801 | Flag-JIP1-PFKFB4 in p3xFLAG-CMV |
| pSH2802 | Flag-JIP1-INPP1 in p3xFLAG-CMV |
| pSH2803 | Flag-JIP1-PSTPIP1 in p3xFLAG-CMV |
| pSH2804 | Flag-JIP1-PHACTR4 in p3xFLAG-CMV |
| pSH2805 | Flag-JIP1-PNKP in p3xFLAG-CMV |
| pSH2806 | Flag-JIP1-PHPT1 in p3xFLAG-CMV |
| pSH2807 | Flag-JIP1-MINPP1 in p3xFLAG-CMV |
| pSH2808 | Flag-JIP1-RNGTT in p3xFLAG-CMV |
| pSH2809 | Flag-JIP1-PTN in p3xFLAG-CMV |

| PTPN1, a novel regulators JNK MAP kinase pathway | |
| --- | --- |
| names | descriptions |
| pSH2066 | pSuper-shRNA of JIP1 (3'UTR: start from 2524) |
| pSH2820 | MLK3-myc/his in pcDNA3.1 |
| pSH2821 | MKK7-myc/his in pcDNA3.1 |
| pSH2822 | JNK-myc/his in pcDNA3.1 |
| pSH2824 | GST-MKK7 in pGEX4T1 |
| pSH2825 | GST-JNK1 in pGEX4T1 |
| pSH2827 | his-MLK3 in pACYC-Duet1 |
| pSH2828 | his-MKK7 CA (S271E T275E S277E) in pACYC-Duet1 |
| pSH2829 | Flag-PTPN1 in p3xFLAG-CMV |
| pSH2830 | Flag-DUSP10 in p3xFLAG-CMV |
| pSH2848 | his-JIP1 in pET-Deut1 |
| pSH2849 | his-MLK3 in pET-Deut1 |
| pSH2850 | his-MKK7 in pET-Deut1 |
| pSH2851 | his-JNK1 in pET-Deut1 |
| pSH2852 | his-MLK3-GST in pET-Deut1 |
| pSH2855 | his-PTPN1 D181A in pET-Deut1 |
| pSH2857 | Flag-PTPN1 D181A in p3xFLAG-CMV |
| pSH2978 | dsRed-JNK in p3xFLAG-CMV |
| pSH2979 | dsRed-JNK-nNOS in p3xFLAG-CMV |

## **Table S2. Phosphatases examined in this study were listed.**

**Listed are 77 human phosphatases that were tested as JIP1-tethered fusion proteins, including DUSPs, protein Ser/Thr phosphatases, protein tyrosine phosphatases and other phosphatases.**

| Phosphatase group | Name | Accession  (protein id) | Size  (bp) | Size  (kDa) |
| --- | --- | --- | --- | --- |
| Protein tyrosine phosphatase | PTPN1 | AAH10191.1 | 1305 | 50.0 |
|  | PTPN2 | NP_033003.1 | 1158 | 45.1 |
|  | PTPN4 | NP_002821.1 | 1512 | 56.9 |
|  | PTPN6 | NP_536859.1 | 1785 | 67.6 |
|  | PTPN7 | NP_002823.3 | 1197 | 45.0 |
|  | PTPN12 | NP_002826.3 | 2340 | 88.1 |
|  | PTPN13 | NP_542416.1 | 1188 | 45.4 |
|  | PTPN14 | NP_005392.2 | 840 | 32.3 |
|  | PTPNS1L2 | NP_542970.1 | 591 | 21.7 |
|  | PTPRB | NP_001103224.1 | 1098 | 42.5 |
|  | PTPRE | NP_006495.1 | 1803 | 69.7 |
|  | PTPRH | NP_002833.3 | 1134 | 43.0 |
|  | PTPRM | NP_001098714.1 | 996 | 38.1 |
|  | PTPRN2 | NP_002838.2 | 1026 | 39.1 |
|  | PTPRO  (transcript variant1) | NP_109592.1 | 1098 | 42.9 |
|  | PTPRO  (transcript variant4) | NP_109593.1 | 1014 | 39.5 |
|  | PTPRT | NP_573400.3 | 1638 | 64.6 |
|  | PTPRS | NP_002841.3 | 1686 | 64.8 |
|  | PTPRZ1 | NP_002842.2 | 1788 | 67.7 |
|  | PTP4A2 | NP_536316.1 | 462 | 17.6 |
|  | PTP4A3 | NP_116000.1 | 516 | 19.4 |
|  | CDC25C | NP_001781.2 | 1419 | 53.3 |
| Dual specificity phosphatases | DUSP3 | NP_004081.1 | 555 | 20.5 |
|  | DUSP4 | NP_001385.1 | 1182 | 43.0 |
|  | DUSP6 | NP_001937.2 | 1143 | 42.3 |
|  | DUSP7 | NP_003575.2 | 960 | 35.3 |
|  | DUSP10 | NP_009138.1 | 1446 | 52.6 |
|  | DUSP11 | NP_003575.2 | 990 | 39.0 |
|  | DUSP12 | NP_009171.1 | 1020 | 37.7 |
|  | DUSP13 | NP_001007274.1 | 594 | 22.1 |
|  | DUSP14 | NP_008957.1 | 594 | 22.3 |
|  | DUSP15 | NP_542178.2 | 705 | 26.2 |
|  | DUSP16 | NP_085143.1 | 1938 | 72.6 |
|  | DUSP18/20 | NP_689724.3 | 564 | 21.1 |
|  | DUSP21 | NP_071359.3 | 570 | 21.5 |
|  | DUSP22 | NP_064570.1 | 171 | 9.5 |
|  | DUSP23 | NP_542178.2 | 450 | 16.6 |
|  | DUSP24 | NP_057170.1 | 939 | 35.8 |
|  | DUSP26 | NP_076930.1 | 633 | 23.9 |
|  | TNS | NP_072174.3 | 855 | 32.5 |
|  | CDKN3 | NP_005183.2 | 498 | 18.7 |
|  | STYX | NP_660294.1 | 669 | 25.8 |
|  | MTMR3 | NP_066576.1 | 1182 | 44.7 |
|  | MTMR8 | AAH12399.1 | 1749 | 65.3 |
|  | PTEN | NP_000305.3 | 1209 | 47.2 |
|  | RNGTT | AAH19954.1 | 1791 | 65.3 |
| Ser/Thr phosphatases | PPA1 | NP_066952.1 | 867 | 32.7 |
|  | PPM1A | NP_808821.2 | 1146 | 42.4 |
|  | PPP1CA | NP_001008709.1 | 990 | 37.5 |
|  | PPP1CB | NP_996759.1 | 981 | 37.2 |
|  | PPP2CA | NP_002706.1 | 927 | 35.6 |
|  | PPP2CB | NM_004156 | 927 | 35.6 |
|  | PPP3CA | NP_000935.1 | 1533 | 57.7 |
|  | PPP4C | NP_002711.1 | 921 | 35.1 |
|  | PPP5C | NP_006238.1 | 1437 | 54.7 |
|  | PPP6C | NP_001116827.1 | 915 | 35.1 |
|  | PPP1R1B | NP_115568.2 | 612 | 23.0 |
|  | PPP1R8 | NP_054829.2 | 627 | 22.7 |
|  | PPP2R1A | NP_055040.2 | 1767 | 65.3 |
|  | PPP2R2A | NP_001171062.1 | 1341 | 51.7 |
|  | PPP2R2B | NP_858062.1 | 1527 | 58.9 |
|  | PPP2R3B | NP_037371.2 | 528 | 20.1 |
|  | PPP2R5D | NP_006236.1 | 1806 | 70.0 |
|  | PPP3R1 | NP_000936.1 | 510 | 19.3 |
| acylphosphatase | ACYP1 | NP_001098.1 | 297 | 11.3 |
| acylphosphatase | ACYP2 | NP_612457.1 | 297 | 11.1 |
| alkaline phosphatase | ALPP | NP_001623.3 | 1605 | 58.0 |
| fructose-1,6-bisphosphatase 1 | FBP | NP_000498.2 | 1014 | 36.8 |
| 6-phosphofructo-2-kinase | PFKFB4 | AAH10269.1 | 1407 | 54.0 |
| proline-serine-threonine phosphatase interacting protein 1 | PSTPIP1 | AAH08602.1 | 1248 | 47.6 |
| phosphatase and actin regulator 4 | PHACTR4 | AAH29266.1 | 2106 | 78.2 |
| polynucleotide kinase 3'-phosphatase | PNKP | AAH33822.1 | 1563 | 57.1 |
| Histidine phosphatase domain | ACP6 | NP_057445.3 | 1248 | 48.8 |
| phosphohistidine phosphatase 1 | PHPT1 | AAH24648.1 | 375 | 13.8 |
| inositol polyphosphate-1-phosphatase | INPP1 | AAH15496.1 | 1197 | 44.0 |
| multiple inositol polyphosphate histidine phosphatase, 1 | MINPP1 | AAH32504.1 | 1461 | 55.1 |
|  | PTN | NP_002816.1 | 504 | 18.9 |
